# What do we know about winter active ground beetles (Coleoptera, Carabidae) in Central and Northern Europe?

**DOI:** 10.3897/zookeys.100.1543

**Published:** 2011-05-20

**Authors:** Radomir Jaskuła, Agnieszka Soszyńska-Maj

**Affiliations:** Department of Invertebrate Zoology & Hydrobiology, University of Łódź, Banacha 12/16, 90-237 Łódź, Poland

**Keywords:** Coleoptera, Carabidae, Central Europe, winter activity, subnivean fauna

## Abstract

This paper summarizes the current knowledge on winter active Carabidae in Central and Northern Europe. In total 73 winter active species are listed, based on literature and own observations. Ground beetles are among the three most numerous *Coleoptera* families active during the autumn to spring period. The winter community of *Carabidae* is composed both of larvae (mainly autumn breeding species) and adults, as well as of epigeic species and those inhabiting tree trunks. Supranivean fauna is characterized by lower species diversity than the subnivean fauna. The activity of ground beetles decreases in late autumn, is lowest during mid-winter and increases in early spring. *Carabidae* are noted as an important food source in the diet of insectivorous mammals. They are also predators, hunting small winter active invertebrates.

## Introduction

During winter, invertebrates are mostly inactive in diapause as eggs, larvae or pupae, but less often as adult stages (Leather et al. 1993). Body fluids may freeze in low temperatures, so to avoid death, insects employ two main strategies: avoiding freezing or tolerating freezing. The adaptation to avoid freezing is the ability of supercooling by synthesizing antifreezing agents (e.g., glycerol) (Moore and Lee 1991). Some poikilothermic organisms may stay active in winter. These organisms use favourable atmospheric condition – mild winter days with low air pressure – for migration and copulation (Soszyńska 2004). Their activity in low temperatures is usually related to the presence of snow and to their thermal properties. Snow cover has a high insulation capacity and low thermal conductivity due to its high air content. Low density and a greater thickness of snow (depending on geographical area) provide better insulation. The soil and litter can remain warm even if air temperature is very low (Aitchison 1974, 2001).

Snow cover provides winter active animals with three different microhabitats. The insulating properties of snow make the space under the snow a favourable habitat for invertebrates (subnivean microenvironment). The subnivean microhabitat is relatively warm, humid, thermally stable and protects organisms from wind and lethal temperatures in contrast to the snow surface (supranivean environment), which is highly variable and completely dependent on atmospheric factors. Within the snow, the so-called intranivean habitat, temperatures are lower but organisms are still protected from the external environment (Aitchison 2001). Animals that are active in snow can be divided into two main groups, depending on their period of activity. The first group consists of “true winter” organisms that are active during the winter months (end of November until the beginning of April) both under and on the snow cover. The second is a nival fauna that are active on the snow cover outside the winter months. Examples of these fauna are permanent residents of high-altitude regions and glaciers. These invertebrates are adapted to permanent snow, glacier surfaces, etc. Their food source is the aeolian fauna, which consists of invertebrates passively deposited on snow fields (Mani 1962, Aitchison 2001).

The snow fauna is an ecological group, which consists of permanent snow active invertebrate species. The first observations regarding invertebrate activity on the snow was made in Poland in the middle of 18th century (Fedorowicz 1968). Since then, snow active insects have been the main subject of investigation in only a few elaborate studies. Snow activity was observed in many insect orders: Collembola, *Trichoptera*, *Plecoptera*, Blattodea, *Hemiptera*, *Mecoptera*, *Coleoptera*, *Diptera*, and *Hymenoptera* (Frey 1913, Tahvonen 1942, Szulczewski 1947, Ulfstrand 1968, Brummer-Korvenkontio and Brummer-Korvenkontio 1980, Leinaas 1981, 1983, Hågvar 1995, 2000, Aitchison 2001, Soszyńska and Durska 2002, Soszyńska 2004, Hågvar and Greve 2003).

The first information about subnivean fauna appeared almost two centuries later than that of the fauna living on the snow. The subnivean microenvironment is inhabited by more numerous groups of invertebrates, such as oligochaetes, molluscs, crustaceans, arachnids and insects. Among these, insects and spiders clearly predominate, being the major representatives of the snow active fauna. The subnivean fauna was studied more often than the snow active fauna. Main studies came from Canada (Aitchison 1978, 1979a-d, 1984, 2001), the USA (Schmidt and Lockwood 1992, Addington and Seastedt 1999), as well as from central and northern Europe (Renken 1956, Ackefors 1964, Näsmark 1964, Merriam et al. 1983, Itämies and Lindgren 1989, Łęgowski and Łoziński 1995). The most common orders in terms of species diversity as well as percentage contribution to this ecological group are Collembola, *Coleoptera*, *Diptera*, but also *Hymenoptera*, and *Hemiptera* (Tahvonen 1942, Ackefors 1964, Aitchison 1979a-d, 1984, 2001).

During the last decades, global climate change has become an important scientific topic. However its influence on poikilothermic organisms has been poorly investigated. It seems that the occurrence of snow cover during the winter period plays an important role in the biology of many different invertebrate groups.

The aim of this paper is to summarize knowledge regarding winter active *Carabidae* fauna from Central and Northern Europe. In the present paper we discuss only the “true winter active” ground beetles, and not members of the nival fauna occurring in high-altitude regions or glaciers.

## Methods

Winter season is defined here as the period between the end of November and the beginning of April. All available literature data on winter active *Carabidae* recorded from Central and Northern Europe were used in this study. In total, data from five countries and published in 17 papers were analyzed (see Table 1). Data of mountain *Carabidae* active on the snow and glaciers as well as species found overwintering in diapause were not included. In addition, our unpublished records of winter active *Carabidae* from Central Poland were included. This material was collected occasionally during different field studies using pitfall traps (subnivean species) and active searching on the snow cover. The list of species analyzed in this study is given in Table 1.

All recorded ground beetle species were divided into three groups, according to the microenvironment in which they were noted: epigeic (subnivean), active on the snow cover (supranivean), and actively walking on tree trunks. Data on activity of both the adults and the larvae are also shown in Table 1.

The ecological response towards snow active ground beetle species was done according to Fudakowski (1959) and Pruitt (1978). These authors distinguished the following species groups according to their ecological reaction towards snow: chionobionts – stenothermic species with adaptations to survive on snow and to reproduce in winter, chionophiles – eurythermic permanent snow active group, but its members occur also in other seasons, chionoxenes – species accidentally found in winter; chionophobes – group that avoids snow.

For the nomenclature of *Carabidae* species, the Fauna Europea Web Service (2004) was followed, while the zoogeographical analysis of ground beetles was based on the study by Leśniak (1988).

## Results and discussion

### Winter active Carabidae – a short history of faunistic studies

Most studies performed on winter active ground beetles are rather recent (Table 1). The first faunistic data on winter active *Carabidae* came from the beginning of 20th century, when five species belonging to the genera *Leistus*, *Bradycellus*, *Dromius*, *Ocydromus* and *Pterostichus* were noted in Finland as active on the snow surface by Frey (1913) and Levander (1913). After more than three decades, one additional species from the genus *Agonum* was found on the snow surface by Polish entomologist Szulczewski (1947) in the Wielkopolski National Park (western Poland). More recently, one additional species – *Nebria brevicollis* – was reported by Jaskuła et al. (2005) from central Poland. All these papers presented only single, accidental observations. Our work summarizes up-to-date knowledge about this ecological group and gives a list of 11 species belonging to 10 genera, including first data on activity of the genera *Calathus*, *Carabus*, *Notiophilus*, and *Paradromius* from the snow surface.

Compared to supranivean species (which are easier to observe because of the contrast between the white colour of the snow and the dark coloured insects), the carabids active under the snow surface (subnivean fauna) were discovered rather late. First data on subnivean ground beetles became available after using Barber’s traps as a collecting method, and in Central and Northern Europe were given from Germany by Renken (1956). He provided information on seven species of *Carabidae* from the following genera: *Anchomenus*, *Demetrias*, *Dicheirotrichus*, *Calathus*, *Nebria*, *Philorhizus* and *Trechus*. All these species were imagines. However Evans (1969), using the same method of study, recorded also larvae of *Cychrus caraboides*, *Nebria brevicollis*, *Abax* sp., *Pterostichus* sp., *Leistus* sp., and *Carabus* sp. as being active under the snow surface. Additional records of *Carabidae* larvae were added by Kaczmarek (1958), Näsmark (1964), Greenslade (1965), and here.

More adult beetles were later collected by Kaczmarek (1958 – 6 species from 6 genera), (Näsmark (1964 – 1 species), Greenslade (1965 –18 species from 12 genera), Murdoch (1967 – 17 species from 12 genera), Flatz and Thaler (1980 – 6 species from 6 genera), Betz (1992 – 1 species), Kennedy (1994 – 11 species from 8 genera), Traugott (1998 – 7 species from 5 genera), and Jaskuła and Grabowski (2003 – 1 species). Finally, in the present paper a list of 66 *Carabidae* species is given, including one genus (*Anisodactylus*) recorded for the first time as a supranivean taxon.

**Figure 1.**
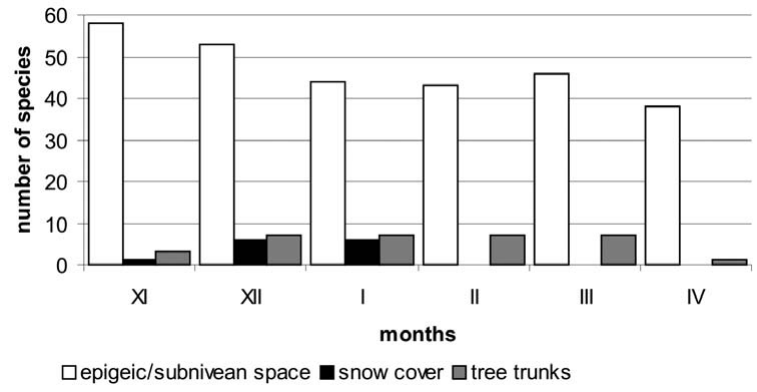
Comparision of subnivean, supranivean and tree trunk fauna of *Carabidae* from Central and Northern Europe during the winter season (based on different sources).

Comparing the two above-mentioned “ecological groups”, it becomes clear that in the studied area, diversity of the subnivean carabid fauna is more than five times higher than that of the supranivean species (Fig. 1). A similar tendency was observed in Collembola, but was opposite when compared to some other insect groups like *Diptera* or *Mecoptera* (Soszyńska-Maj 2005).

Tree trunks are the third type of microhabitat where winter active *Carabidae* occur. The only paper on this topic known to us comes from Hannig et al. (2006) who noted six species in Germany: *Amara familiaris*, *Calodromius bifasciatus*, *Calodromius spilotus*, *Philorhizus melanocephalus*, *Dromius angustus*,and *Dromius quadrimaculatus*. Among them, *Dromius quadrimaculatus* predominated and the genus *Calodromius* was noted as winter active for the first time (see Felix and Van Wielink 2011).

**Table 1.** List of winter active ground beetles (**A** – adults, **L** – larvae). Roman letters indicate the month(s) of observation(s). Nomenclature after Fauna Europaea Web Service (2004).

*No.*	*Species*		*Snow cover*	*Epigeic(subnivean)*	*Treetrunks*	*Source*
1	*Abax parallelepipedus* (Piller et Mitterpacher, 1783)	A		XI-XII, III-IV		Greenslade 1965, Murdoch 1967
2	*Abax* sp./*Pterostichus* sp.	L		XI-XII		Evans 1969
3	*Acupalpus dubius* Schilsky, 1888	A		XII		Murdoch 1967
4	*Agonum gracile* Sturm, 1824	A		IV		this paper
5	*Agonum muelleri* (Herbst, 1784)	A	I			Szulczewski 1947
6	*Agonum viduum* (Panzer, 1796)	A		XI		Murdoch 1967
7	*Amara aulica* (Panzer, 1796)	L		XI-I		Traugott 1998
8	*Amara brunnea* (Gyllenhal, 1810)	A		XI-XII		this paper
9	*Amara communis* (Panzer, 1797)	A		XI-XII		Flatz and Thaler 1980, this paper
10	*Amara infima* (Duftschmid, 1812)	A		XI-I (?)		Kaczmarek 1958
11	*Amara familiaris* (Duftschmid, 1812)	A			III-IV	Hannig et al. 2006
12	*Amara lunicollis* Schiødte, 1837	A		III-IV		Greenslade 1965
13	*Amara* sp.	A		XI-IV		Kennedy 1994
14	*Anchomenus dorsalis* (Pontoppidan, 1763)	A		XI-IV		Renken 1956, Greenslade 1965, Weber 1965, Flatz and Thaler 1980
15	*Anisodactylus binotatus* (Fabricius, 1787)	A		IV		this paper
16	*Asaphidion flavipes* (Linné, 1761)	A		XI-IV		Weber 1965, Murdoch 1967
17	*Asaphidion pallipes* (Schrank, 1781)	A		IV		this paper
18	*Badister sodalis* (Duftschmid, 1812)	A		III-IV		Murdoch 1967
19	*Bradycellus caucasicus* (Chaudoir, 1846)	A	I	XI-I		Frey 1913, Kaczmarek 1958
20	*Bradycelus harpalinus* (Audinet-Serville, 1821)	A		XI-XII		this paper
21	*Bradycellus verbasci* (Duftschmid, 1812)	A		XI-XII, II-III		Evans 1969, this paper
22	*Calathus erratus* (C.R. Sahlberg, 1827)	A		XI		Renken 1956, Kaczmarek 1958
23	*Calathus fuscipes* Goeze, 1777	A		XI-IV		Greenslade 1965, Flatz and Thaler 1980, Kennedy 1994, this paper
		L		XI-IV		Traugott 1998
24	*Calathus melanocephalus* (Linné, 1758)	A		XI-IV		Greenslade 1965, Kennedy 1994
		L		XII-I		Traugott 1998
25	*Calathus micropterus* (Duftschmid, 1812)	A	XI	XI-I (?)		Kaczmarek 1958, this paper
26	*Calathus rotundicollis* Dejean, 1828	A		XI-XII		Greenslade 1965
27	*Calodromius bifasciatus* (Dejean, 1825)	A			XI-III	Hannig et al. 2006
28	*Calodromius spilotus* (Illiger, 1798)	A			XI-III	Hannig et al. 2006
29	*Carabus convexus* Fabricius, 1775	A		XI-III		this paper
30	*Carabus coriaceus* Linné, 1758	L		XII-I		Traugott 1998
31	*Carabus hortensis* Linné, 1758	L		XII		Traugott 1998
32	*Carabus nemoralis* O. F. Müller, 1764	A	I	XI-IV		Greenslade 1965, Weber 1965, Evans 1969, Kennedy 1994, this paper
33	*Carabus problematicus* Herbst, 1786	A		XI		Greenslade 1965, Evans 1969, Betz 1992
34	*Carabus* sp.	L		XI-III		Evans 1969
35	*Cychrus caraboides* (Linné, 1758)	L		XI-XII, III		Evans 1969
36	*Demetrias atricapillus* (Linné, 1758)	A		+		Renken 1956
37	*Dicheirotrichus cognatus* (Gyllenhal, 1827)	A		+		Renken 1956
38	*Dicheirotrichus placidus* (Gyllenhal, 1827)	A		XII		Murdoch 1967
39	*Dromius angustus* Brullé, 1834	A			XII	Hannig et al. 2006
40	*Dromius quadrimaculatus* (Linné, 1758)	A			XI-III	Hannig et al. 2006
41	*Dromius schneideri* Crotch, 1871	A	I			Frey 1913
42	*Dyschiriodes globosus* (Herbst, 1784)	A		XI-IV		Weber 1965
43	*Elaphrus cupreus* Duftschmid, 1812	A		III		Murdoch 1967
44	*Epaphius secalis* (Paykull, 1790)	A		XI-I (?)		Kaczmarek 1958
45	*Leistus rufomarginatus* (Duftschmid, 1812)	A		XI, I-II		Jaskuła and Grabowski 2003, this paper
46	*Leistus ferrugineus* (Linné, 1758)	A		XI-I (?)		Kaczmarek 1958
		L	I	XI-XII, III-IV		Levander 1913, Näsmark 1964, Greenslade 1965
47	*Leistus fulvibarbis* Dejean, 1826	A		XI-XII		Murdoch 1967
48	*Leistus terminatus* (Panzer, 1793)	A		XI-II		Murdoch 1967
		L		XI-IV		Murdoch 1967
49	*Leistus* sp.	L	XII	II-III		Evans 1969, this paper
50	*Loricera pilicornis* (Fabricius, 1775)	A		XI-IV		Greenslade 1965, Murdoch 1967, Kennedy 1994, this paper
51	*Metallina lampros* (Herbst, 1784)	A		XI-IV		Greenslade 1965, Kennedy 1994, this paper
52	*Nebria brevicollis* (Fabricius, 1792)	A		XI-IV		Renken 1956, Greenslade 1965, Murdoch 1967, Evans 1969, Flatz and Thaler 1980, this paper
		L		XI-IV		Murdoch 1967, Evans 1969, Traugott 1998
53	*Notiophilus biguttatus* (Fabricius, 1779)	A	XII-I	XI-IV		Greenslade 1965, Evans 1969, Kennedy 1994, this paper
54	*Notiophilus rufipes* Curtis, 1829	A		XI, I-II		Greenslade 1965
55	*Notiophilus substriatus* C.R. Waterhouse, 1833	A		XI, I-II		Greenslade 1965
56	*Ocydromus tetracolus* (Say, 1823)	A	XII	XI-IV		Frey 1913, Murdoch 1967, Weber 1965, Kennedy 1994
57	*Panagaeus bipustulatus* (Fabricius, 1775)	A		IV		this paper
58	*Paradromius linearis* (Olivier, 1795)	A	XII	XII		Murdoch 1967, this paper
59	*Paranchus albipes* (Fabricius, 1796)	A		XI, II-IV		Murdoch 1967
60	*Philochthus aeneus* (Germar, 1824)	A		XI-IV		Kennedy 1994
61	*Philochthus biguttatus* (Fabricius, 1779)	A		XI-IV		Murdoch 1967
62	*Philochthus guttula* (Fabricius, 1792)	A		XI, I-IV		Murdoch 1967
63	*Philorhizus melanocephalus* (Dejean, 1825)	A		+	XII	Renken 1956, Hannig et al. 2006
64	*Phyla obtusa* (Audinet-Serville, 1821)	A		XI-IV		Weber 1965, Kennedy 1994
65	*Poecilus versicolor* (Sturm, 1824)	A		XI, I,II		Greenslade 1965, Flatz and Thaler 1980
66	*Pseudoofonus rufipes* (De Geer, 1774)	A		XI-IV		Greenslade 1965, Weber 1965
		L		XI-III		Traugott 1998
67	*Pterostichus diligens* (Sturm, 1824)	A	XII	IV		Frey 1913, this paper
68	*Pterostichus madidus* (Fabricius, 1775)	A		XI-IV		Greenslade 1965, Murdoch 1967
69	*Pterostichus melanarius* (Illiger, 1798)	A		XI-IV		Weber 1965, Flatz and Thaler 1980, Kennedy 1994
		L		XI-I		Traugott 1998
70	*Pterostichus niger* (Schaller, 1783)	A		IV		this paper
71	*Pterostichus nigrita* (Paykull, 1790)	A	XII	XI-XII, II-IV		Murdoch 1967, this paper
72	*Pterostichus oblongopunctatus* (Fabricius, 1787)	A		XII, III-IV		this paper
73	*Pterostichus quadrifoveolatus* Letzner, 1852	A		XI-XII		Kaczmarek 1958
74	*Pterostichus strenuous* (Panzer, 1796)	A		XI-IV		Murdoch 1967, Evans 1969
75	*Pterostichus* sp.	L		XI-IV		Weber 1965
76	*Stomis pumicatus* (Panzer, 1796)	A		III		Murdoch 1967
77	*Trechus obtusus* Erichson, 1837	A		XI-XII, III		Murdoch 1967
78	*Trechus quadristriatus* (Schrank, 1781)	A		XI-IV		Renken 1956, Weber 1965, Kennedy 1994
79	Larvae gen. sp.	L		XI-IV		Renken 1956, Kaczmarek 1958, this paper
*TOTAL*			*11*	*66*	*6*	

### Winter active carabid communities

The most common groups among winter active invertebrates are spiders and insects. Among hexapods, springtails (Collembola), beetles (*Coleoptera*), flies (*Diptera*) and scorpionflies (*Mecoptera*) predominate. Beetle activity under snow cover is well documented. Investigations on winter active fauna in central Poland show that the supranivean and subnivean insect winter assemblages differ in terms of percentage contribution of orders, as well as in species composition. Beetles have only a share of 13% in snow active insect communities, and 25% in material collected under the snow (Soszyńska-Maj 2005). Among subnivean *Coleoptera*, three families clearly predominate: *Staphylinidae*, *Carabidae* and *Cantharidae* (larvae), while carabids are only found accidentally on the snow. These three beetle groups are known as winter dominants, both in terms of species diversity and abundance (Wolska 1957, Strübing 1958, Renken 1956, Näsmark 1964, Weber 1965, Aitchison 1979b, 1984, 2001, Merriam et al. 1983, Itämies and Lindgren 1989, Łęgowski and Łoziński 1995, Traugott 2002). A total of 16 *Coleoptera* families have thus far been recorded as winter active (Hannig et al. 2006, Soszyńska-Maj and Jaskuła unpublished data).

In general, the winter activity of Carabidae varies seasonally. Its peak – both according to the number of species and individuals – is observed in late autumn and early spring. The lowest activity is observed in mid-winter (Fig. 1). Current analysis suggests that the diversity of ground beetles that are active under the snow cover is even several times higher than in supranivean fauna. The number of subnivean species active during the winter can be similar for months, whereas supranivean carabids occur more accidentally. As can be seen from Table 1, only a few species are regularly observed as being active during the whole winter and from many regions. For most species described as winter active only one observation of a single individual is recorded. A good example comes from a study by Kennedy (1994) who recorded, between 22 November and 4 April, at least 12 *Carabidae* species (genus *Amara* was provided with no details about species number) in winter-wheat fields in Ireland. Among these percentages, only the proportion of *Phyla obtusa* was higher than all other recorded species – more than 70% of the caught individuals. Only three other species (*Metallina lampros*, *Philochthus aeneus* and *Trechus quadristriatus*) had a share higher than 5%. Similar results came from Murdoch (1967) who recorded 17 species. In this case only *Nebria brevicollis* and *Leistus terminatus* were caught in ‘high’ proportions: respectively 68,9% and 9,1%. *Nebria brevicollis* also clearly predominated among 18 ground beetle species found in winter by Greenslade (1965). Dominance of only single species was noted by Flatz and Thaler (1980; *Poecilus versicolor*) and Hanning et al. (2006; *Dromius quadrimaculatus*). All these results suggest that also among the epigeic ground beetle fauna some species are found occasionally, while at least several others can be classified as permanently winter active.

According to literature data, winter active carabid species are known both from forests and open habitats as well as from species living on tree trunks (Table 1). Moreover, Kennedy (1994) showed that at least some carabid species can be active during the winter period both during night and day. From these investigations it became clear that *Phyla obtusa* was a day active species from 2nd to 24th of January; unfortunately no data about temperature or other environmental factors were given.

In general, *Carabidae* can be divided into two main breeding groups: autumn breeders (eggs are laid during the last weeks of summer and first weeks of autumn) and spring breeders (eggs are laid from March to May). As a result of this division, winter and summer carabid larvae can be distinguished (Luff 1993). Usually winter larvae hatch from from September to November, and can be found (instars 1–3) throughout the winter and in the following spring period. Although the total number of *Carabidae* species that breed in the autumn period is much higher, at the moment larvae of 12 different species have been distinguished as winter active (Levander 1913, Näsmark 1954, Renken 1956, Kaczmarek 1958, Greenslade 1965, Weber 1965, Murdoch 1967, Evans 1969, Luff 1993**,** Traugott 1998, this paper; Table 1). Among these, the occurrence of larvae of *Amara aulica*, *Calathus melanocephalus*, *Carabus coriaceus*, *Carabus problematicus*, *Nebria brevicollis*, *Pseudoophonus rufipes*, *Pterostichus melanarius*, and *Leistus* species can be explained as a result of autumn breeding (Betz 1992, Luff 1993, Traugott 1998). Weber (1965) and Evans (1969) did not provide any details on the identity of the *Carabus* and *Pterostichus* larvae found during the winter period. However, these genera do have species that belong to autumnal breeders too (Luff 1993).

Luff (1993**)** suggested that winter larvae of ground beetles must survive not only low temperatures and food shortages, but also a long period of exposure to natural enemies, and possible flooding. He also noted that, especially at lower temperatures, some winter carabid larvae can survive without food for up to 30 days.

**Figure 2.**
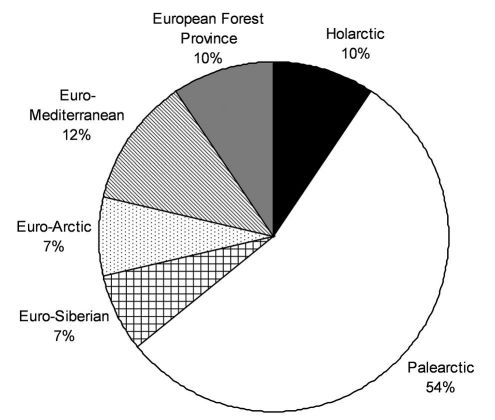
The relative zoogeographical structure of winter active *Carabidae* (based on Leśniak 1988).

A zoogeographical analysis shows that the Central and northern European winter active *Carabidae* most frequently belong to the Palaearctic fauna (54%). Interesting is that Euro-Siberian and Euro-Arctic species (groups that should be adapted evolutionary to low temperatures) made up only 14% of the recorded ground beetle species, while 12% of the species belong the Euro-Mediterranean fauna (Fig. 2).

### Role of carabids in the winter food chain

High densities per square meter and high percentages of *Carabidae* in winter active insect communities make this group an important source of food for insectivorous vertebrates, particularly shrews. Due to their very high metabolic rate these mammals must feed almost constantly to stay alive. They are active all year round, without a hibernation period in winter and their food requirement is 43% higher in winter than in summer (Randolph 1973). As indicated in the literature, shrews do not feed on hibernating invertebrates, but rather on winter-active species, including *Carabidae* (Ackefors 1964, Pernetta 1977, Aitchison 1984, Itämies and Lindgren 1989). Ground beetles can be an attractive type of food for these mammals as they are present in relatively high densities - up to 23 individuals per square meter (Kennedy 1994). Rudge (1968) found that the percentage frequency of beetles in the diet of *Sorex araneus* varies from 66–72% in the autumn and spring. This increases up to 84% in the winter months. In this study only plants had a higher share during the autumn-spring period (percentage frequency 96–100%) and other small vertebrates in autumnal months (100%).

From the winter active Central and North European group of *Carabidae*, 79% of the species appear to be predators (Table 1). Among them there are both large zoophagous species hunting for various types of prey (e.g., *Carabus* species) and specialists collecting small but very abundant prey items, i.e., springtails and aphids (e.g., Aitchison 1978, 1979, Leinaas 1981, 1983, Hågvar 2000). Springtails are known as one of the most abundant subnivean invertebrate groups (e.g., Näsmark 1964, Aitchison 1984). They were regarded as an important food source in the winter active *Phyla obtusa* with a percentage frequency from 4 to 20% (Kennedy 1994). In the latter study *Phyla obtusa* was also noted as a predator of mites (8–30%) and aphids (4–33%) during January-March. Among winter active carabids, species belonging to the genera *Loricera*, *Notiophilus*, *Leistus*, and some smallspecies of *Pterostichus* are also well known as predator of springtails. Most probably the winter activity of species belonging to *Dromius* s.l. (e.g., Hannig et al. 2006) group can be related to the activity of their usual type of prey, i.e., aphids. On the other hand, winter activity of omnivorous (5%) and phytophagous (16%) carabids can be explained by a relatively easy access to their food, i.e., dry or decaying wood, fungi, leaves and seeds.

Many *Carabidae* species can change their diet according to the availability of food in the environment. Some predatory beetles (e.g., some *Carabus* species, *Pterostichus melanarius*, *Calathus fuscipes*, *Nebria brevicollis*) occasionally eat plant material. Also some typically phytophagous species (*Amara* spp., *Harpalus* spp., *Bradycellus* spp.) are able to change their diet to eggs and pupae of flies (Tischler 1971). When temperatures become too low, some species can stop feeding even if they are still active (Aitchison 2001). In extreme situations some beetles (including larvaal stages) can survive up to one month without food while remaining active (Luff 1993).

An important adaptation that protects winter active arthropods from freezing is non-feeding behaviour during lower temperatures (Aitchison 1987). The presence of food in the gut significantly increases the possibility of spontaneous freezing as ice nucleators are present in the food (Salt 1968). As a special adaptation to prevent freezing during eating at cold temperatures, external digestion can be seen in some *Carabidae* species, including members of *Carabus*, *Cychrus*, *Pterostichus*, and ground beetle larvae (Hengeveld 1980a-b, Evans and Forsythe 1985). As was shown by Aitchison (1987), who studied spiders, a group that feeds by means of external digestion, such behaviour allows the avoidance of consuming dust particles on which spontaneous ice formation can occur. One of the most common and abundant groups of winter active arthropods is Collembola, which is also a popular type of prey for some *Carabidae* species. The study of Aitchison (1978) showed that springtails contain some cryoptotectans in their haemolymph allowing survival in cold temperatures. Feeding mechanisms observed in spiders and carabids suggest that these chemical compounds can possibly be transferred from a prey to a predator body during eating. As a result cryoprotectans of the prey may allow its predator to survive low temperatures.

### Weather conditions and winter activity of ground beetles

Based on literature data we can assume that the activity of *Carabidae* species decreases in late autumn. Activity will be lowest during the winter period, and increases in the early spring (e.g., Evans 1969, Table 1, Fig. 1). The subnivean environment is characterized by a much higher number of carabid species compared to the supranivean one (Fig. 1). This is observed in many other insect groups and is usually explained by the role of thermally isolated snow cover that protects the environment from wind and lethal temperatures (Aitchison 2001). Literature data and our own results show that *Carabidae* are active on the snow surface only from November to January, while subnivean activity occurred during the entire winter season. In general, ground beetles are only accidentally found on the snow cover and because of this, they should be classified as chionoxenes.

In the literature there are almost no data on the effects of weather factors on winter active *Carabidae*. A study by Weber (1965) suggests that air temperature rising from -2°C to +6°C increased activity of *Phyla obtusa* almost eight times. Similar observations were made by the same author for *Trechus quadristriatus*.

Interesting observations were made by Haning et al. (2006), who noted *Calodromius bifasciatus* to be active on tree trunks at -3°C and from -1 to +10°C, with males preferring lower temperatures than females (see also Felix and Van Wielink 2011). For supranivean active carabids, temperature data are known for only four species: *Dromius schneideri* was found at *-*1°C, *Pterostichus diligens* at +1°C(Frey 1913), *Agonum muelleri* at +2°C (Szulczewski 1947) and *Calathus micropterus* at -2°C (Soszyńska-Maj & Jaskuła unpublished).

## Conclusions

Present knowledge on winter active *Carabidae* from Central and Northern Europe is rather poor. Literature data are mostly from a few old papers, and usually were fragmentary. In total, 73 species have been recorded as active in winter, including 11 species belonging to 10 genera found on the snow surface, and 66 species from33 genera being subnivean. Four species were recorded for the first time as snow active and one as a subnivean carabid.

Ground beetles are one of the dominating *Coleoptera* groups in winter insect assemblage. The community of winter active *Carabidae* is composed of larvae and adult beetles, and consists of both epigeic species and species active on tree trunks. In general, winter active larvae are representatives of autumn breeders. A comparison of the supranivean and subnivean carabid fauna shows significant differences in species diversity. In the first group the number of species are five times lower than in the latter. It suggests that snow active species appear in supranivean microhabitats only accidentally, but they are known to be winter active in litter or soil environments. They should probably be classified as chionoxenes.

Winter activity of ground beetles decreases in late autumn, is lowest during mid-winter and increases in early spring. This might be correlated with weather conditions, especially air temperature. The present state of knowledge suggests that further studies are needed to confirm this hypothesis.

The high proportion of *Carabidae* in winter communities make this group an important food source in the diet of insectivorous mammals, especially shrews. On the other hand these carabids are predators, hunting springtails and other small winter active invertebrates.
